# Exploring stress and morphology in two songbird species across urban, agricultural, and natural habitats on San Cristobal Island, Galapagos

**DOI:** 10.1186/s40850-025-00221-7

**Published:** 2025-03-10

**Authors:** Andrés Mena, Martín Terán, Diana Calderón, Maria de Lourdes Torres, Diego F. Cisneros-Heredia

**Affiliations:** 1 Laboratorio de Zoología Terrestre, Instituto de Biodiversidad Tropical IBIOTROP, Colegio de Ciencias Biológicas y Ambientales, Universidad San Francisco de Quito USFQ, Quito, 170901 Ecuador; 2 Laboratorio de Biotecnología Vegetal, Colegio de Ciencias Biológicas y Ambientales, Universidad San Francisco de Quito USFQ, Quito, Ecuador; 3 Universidad San Francisco de Quito USFQ, Extensión Galápagos GAIAS, & Galápagos Science Center (Universidad San Francisco de Quito USFQ and University of North Carolina at Chapel Hill UNC), Puerto Baquerizo Moreno, San Cristóbal, Galápagos Ecuador; 4 Laboratorio de Salud Animal, Instituto de Biodiversidad Tropical IBIOTROP, Escuela de Medicina Veterinaria, Universidad San Francisco de Quito USFQ, Quito, Ecuador

**Keywords:** Corticosterone, Galapagos Islands, *Geospiza fuliginosa*, Land use change, Land birds, Morphological adaptations, *Setophaga petechia aureola*, Wildlife stress response

## Abstract

Land use changes can have morphological and physiological impacts on wildlife. This study aimed to explore the influence of anthropogenic land use on the morphology and corticosterone concentrations in two songbirds endemic to the Galapagos archipelago: the granivorous Small Ground Finch *Geospiza fuliginosa* and the insectivorous Galapagos Yellow Warbler *Setophaga petechia aureola* in San Cristobal Island. Birds were caught and measured between June and August 2018 and June and July 2019 across four areas with different human land uses: urban green areas in the coastal town of Puerto Baquerizo Moreno, natural deciduous forest in the lowlands, agricultural areas in the highlands, and seasonal evergreen forest in the highlands. Morphological comparisons among study areas were made using ANOVA or the Kurskall-Wallis test. Corticosterone levels obtained from tail feathers were measured with an ELISA test. Linear regression models were employed to explore the effects of the different human land uses on corticosterone concentrations. For *G. fuliginosa*, we found significant differences (*p* < 0.05) in weight, wing, and tarsus length between natural and disturbed habitats. The linear regression results showed higher corticosterone concentrations in urban *G. fuliginosa* than those in agricultural and natural habitats. Additionally, higher corticosterone concentrations were found in finches captured in 2018, a year with much higher precipitation than in 2019. For *S. petechia aureola*, the only significant difference (*p* < 0.05) between areas was a wider beak in birds captured in the seasonal forest compared to those from urban areas. Although our sample size does not allow for definitive conclusions, our results provide evidence that the ecology of each species plays a crucial role in shaping their morphological and physiological responses to land use changes and seasonal environmental changes.

## Introduction

Natural ecosystems are undergoing drastic transformations due to the intensification of urban and agricultural land use [1, 2]. These human-induced disturbances can destabilise local animal communities by altering the availability of food, breeding sites, and hunting territories [3–6]. In addition to ecological impacts, land use changes may also act as stressors, leading to morphological and physiological consequences for animals [7–9]. Stress may lead to changes in the size and shape of individuals [10–12] while also modifying their levels of glucocorticoids—hormones that modulate animal response to new environmental demands [7]. While short-term glucocorticoid secretion can enhance fitness in critical situations [7], prolonged elevated levels may impair physiological homeostasis [7, 13, 14].

Birds are an effective animal model for hormonal studies because they have been extensively studied, and robust methodologies and extensive reference data are available [15, 16]. Most studies of avian stress in relation to human impact use the hormone corticosterone as an indicator [15]. Corticosterone is a physiological marker released along with other glucocorticoids in the stress response mediated through the hypothalamic-pituitary-adrenal axis [14, 17]. Previous reports in birds have found contrasting results regarding changes in corticosterone levels due to urbanisation [15, 18–22]. For certain species, differences between hormone concentrations between urban and non-urban bird populations have been suggested [15, 19, 23]. Given the potential adverse effects of prolonged corticosterone elevation on metabolism and the species-specific nature of hormonal responses, it is essential to account for interspecific variation when studying stress responses in avian communities under human disturbance.

Oceanic islands are ecosystems that are notably vulnerable to land-use changes, primarily due to their high degree of endemism and limited geographic range. These islands are particularly prone to biodiversity loss due to habitat changes [24, 25]. A concerning example is Hawaii, where over half of the native birds have become extinct due to human-related impacts [26]. In this context, the inhabited islands of the Galapagos archipelago offer a suitable scenario for studying the effects of human land use on bird populations as healthy native habitats converge with disturbed environments [27–29]. Since 1959, 97% of the archipelago area has been protected under the Galapagos National Park, and human land use is mainly confined to only 3% of the territory. Urban centres and agricultural areas on the inhabited islands of the Galapagos directly border large areas of protected native ecosystems, lacking a vegetational cover gradient [28, 29]. The boundaries of a national park are inherently artificial, and animals and plants frequently disperse over the border between the national park and the designated urban and agricultural areas [30].

Unlike other oceanic islands, the inhabited islands of the Galapagos have not experienced mass extinctions; however, there is growing concern over declines in endemic bird populations [31, 32]. Reported alterations in the morphology, gut microbiota, and even epigenetic changes due to anthropogenic land-use expansion and intensification have been observed in populations of Darwin’s finches in the Galapagos [33–37]. Other species are also affected by anthropogenic disturbance, including the Galapagos Yellow Warbler, *Setophaga petechia aureola*, which is one of the most impacted species by roadkill [38]. Changes in land use, both agricultural and urban, may influence the spread dynamics of avian diseases such as avian pox (*Avipoxvirus* spp.) and avian malaria (*Plasmodium* spp.) circulating on the human-inhabited islands of the Galápagos [39–41].

To the best of our knowledge, most studies of bird populations exposed to anthropogenic impacts on oceanic islands have focused on species richness, abundance, or habitat use, with few addressing questions related to physiological changes [41–43]. This study aims to explore the effects of urban and agricultural land use on the morphology and corticosterone levels of two songbird species, the granivorous Small Ground Finch *Geospiza fuliginosa* and the insectivorous Galapagos Yellow Warbler *Setophaga petechia aureola*, on San Cristobal Island, Galapagos. We hypothesise that these birds will exhibit differences in hormone concentrations and morphological traits between natural, agricultural, and urban environments on San Cristobal Island, given the marked changes in available resources in those environments.

## Methodology

### Study area

The Galapagos Archipelago comprises 19 main islands (area > 1 km^2^) and over 100 islets and rocks, with a total area of 7850 km^2^. These volcanic marine islands are situated 930 km off the coast of Ecuador [44]. The climate of the Galapagos is heavily influenced by oceanic currents and winds, with vegetation distribution primarily determined by orogenic rainfall [45, 46]. The islands of Santa Cruz, San Cristobal, Isabela, and Floreana are inhabited by humans, with an estimated population of 33,042 people living in the archipelago as of 2020 [47]. We carried out this study on San Cristobal, the easternmost island of the archipelago [44, 48]. San Cristobal, the fifth-largest island with an area of 558 km^2^, is also the geologically oldest island in the Galapagos [44, 49].

### Ethics statement

Our study was authorised under research permits PC-51-18 and PC-59-19, issued by the Galapagos National Park Directorate (Dirección del Parque Nacional Galápagos, Ministerio del Ambiente). We followed the guidelines for using wild birds in research by Fair et al. [50].

### Study design and data collection

**Fig. 1:**
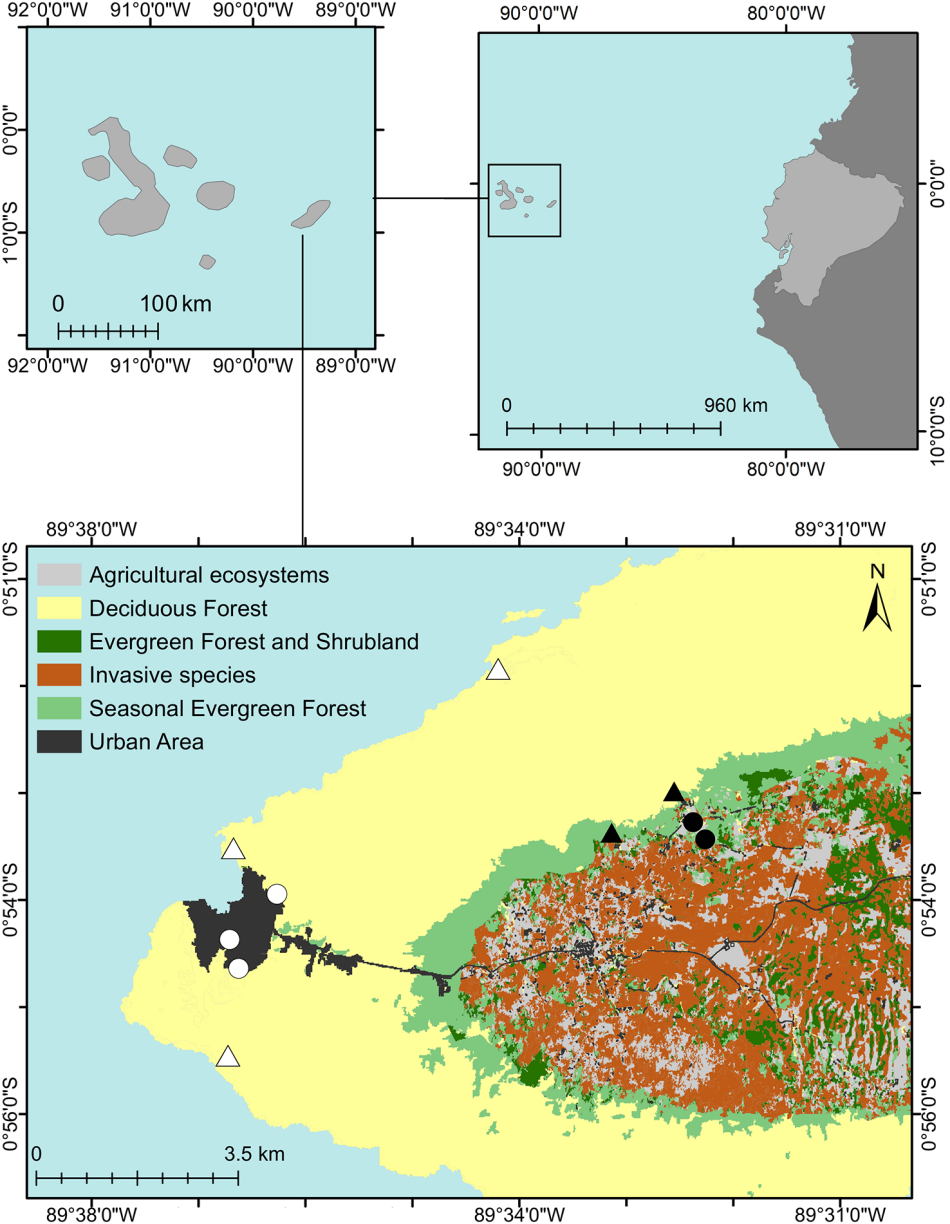
Map of San Cristobal Island, Galapagos archipelago, showing the main habitats and sampling points. Black triangles represent sampling points in Seasonal Evergreen Forest; black circles, sampling points in Agricultural Areas; white circles, sampling points in Urban Areas; and white triangles, sampling points in Deciduous Forest

This study was conducted as part of a monitoring programme of the land bird populations on San Cristobal Island. We focused on analysing data from *Geospiza fuliginosa* and *Setophaga petechia aureola* because they were the most frequently captured species during our mist-net surveys. The different feeding guilds of these songbirds suggest that human impacts would differ between the granivorous *G. fuliginosa* and the insectivorous *S. petechia aureola*. Both taxa are endemic to the Galapagos and are not currently considered threatened by extinction (Least Concern, Freile et al. [32]). Surveys were conducted for 36 days in 2018 (from 21 June to 08 August) and 30 days in 2019 (from 07 June to 17 July) in four habitats chosen for their different degrees of human land use (Table 1, Fig. 1), including urban areas, agricultural areas, deciduous forests, and seasonal evergreen forests. We sampled two or three points within each habitat. At each sampling point, we captured land birds using three mist nets in vegetation gaps. We used two 6 × 2.6 m mist nets and one 12 × 2.6 m mist net. Mist nets were placed in the same position within the same year, but their position varied between years. The placement and orientation of the mist nets were chosen according to the recommendations of Ralph et al. [51]. For each captured bird, we recorded the species identity and five morphometric measurements: tarsus length, wing length, beak width, beak length, and beak depth. Two tail feathers were collected from each individual for corticosterone extraction. To track recaptures and minimise handling time, each captured bird was marked on the tarsus with nail polish prior to release. Sampling occurred continuously during morning and afternoon sessions, except on days of heavy rainfall. During our study, we did not detect any movement of marked birds between our sampling areas. Flight distances between these areas ranged from 0.8 to 6 km. Previous research has indicated that *G. fuliginosa* does not typically disperse across different habitats [52, 53]. It is unlikely that finches and warblers involved in our study foraged across the various study areas.

**Table 1:** Land cover description of the four sampling areas on San Cristóbal Island, Galapagos, surveyed during the study

Surveyed habitat	Sampling points	Description
Urban area	Three sampling points in green urban areas of the town of Puerto Baquerizo Moreno, lowlands of San Cristobal Island.	The coastal town of Puerto Baquerizo Moreno covers an area of 7.5 km^2^ surrounded by deciduous forests that are within the Galapagos National Park. Most of the town’s nearly 7000 inhabitants reside in a concentrated urban area of 1.53 km^2^ [54]. Urban green areas are situated adjacent to residential neighbourhoods, often along the town borders, and feature a combination of native and non-native xeric scrub vegetation.
Agricultural area	Two sampling points in silvopasture areas in the highlands of San Cristobal Island.	The agricultural area is in the humid highlands of the island. Our sampling sites were in a silvopasture landscape [28, 29] covered by cultivated grasses for cattle, predominantly *Brachiaria* or *Paspalum*, interspersed with *Citrus* and *Psidium guajava* trees. These points were located a few kilometres away from the border of the national park.
Deciduous forest	Three sampling points at the localities of Playa Carola, La Lobería and Playa Ochoa, lowlands of San Cristobal Island.	These sampling points were in the arid zone of the island, where deciduous scrub and forest grow on lava soils [28, 29]. The vegetation was dominated by native species, such as *Vachellia* spp., *Bursera graveolens, Piscidia carthagenensis, Croton scouleri*, and *Opuntia* spp. These three points were in touristic beach trails, two close to the town of Puerto Baquerizo Moreno, Playa Carola and Playa Loberia, and a more distant and less touristic beach, Playa Ochoa.
Seasonal evergreen forest	Two sampling points in forested remnants on the highlands of San Cristobal Island.	These sampling points were in the humid zone of the island, where cloud-forest-type vegetation grows in small patches preserved by the national park [28, 29]. The habitat includes native species such as *Volkameria mollis, Cordia lutea, Chiococca alba, Psidium galapageium* and *Tournefortia* spp., usually within a matrix with non-native species.

### Corticosterone extraction from feathers

Feathers from *G. fuliginosa* and *S. petechia aureola* were stored in dry paper bags for up to one year before processing. Corticosterone was extracted from feathers following the protocol described by Bortolotti et al. [55], with modifications due to equipment availability and the smaller size of our feathers. Specifically, we suspended our samples in 5 ml of methanol (instead of 10 ml) and employed a different method of methanol extraction after sonication, as explained below.

After removing the calamus with sterilised surgical scissors, each feather was weighed and cut into pieces smaller than 5 mm^2^. Subsequently, 5 mL of methanol were added, and the mixture was immediately sonicated for 30 min. Samples were incubated overnight at 50 °C. For *G. fuliginosa* feathers, the corticosterone-methanol solution was separated from the feather solid material using a graduated pipette. For *S. petechia aureola*, a syringe filter with a 0.45 µm pore size hydrophilic PVDF membrane was used. In both cases, the tubes used for sonication were rinsed with 2.5 mL of methanol to recover as many hormones as possible. The samples were incubated at 50 °C until total methanol evaporation. The extracted residues were reconstituted in a small volume of PBS, ranging between 1–2.5 mL for *G. fuliginosa* samples and 1.5 mL for *S. petechia* samples. Samples in PBS were stored at −20 °C until further analysis.

### Measurements of corticosterone concentrations

Corticosterone concentrations were measured using an enzyme-linked immunoadsorption assay (ELISA) with the Cortisol Labor Diagnostika Nord kit LDN®. The kit allows corticosterone detection by cross-reactivity, measuring relative amounts of corticosterone content in each sample and allowing for measurement comparison to establish tendencies. The protocol followed the manufacturer’s instructions. Hormone concentration results were automatically obtained using Thermo Scientific TM MultiSkan TM SkanIt Software 5.0 for microplate readings. The standard curve was adjusted using a four-parameter logistic regression (4-PL). Results per sample were corrected for feather weight and the PBS suspension volume, resulting in measurements in ng of cortisol/mg of feather. Assays were validated following precision criteria, using the coefficient of variation (CV). Precision was evaluated by setting an intra-assay variability limit of 20% as acceptable [56]. All subsequent morphometric and corticosterone regression model statistical analyses were performed using R software (version 4.04). For Dunn test analysis, we used the package ‘dunn.test’ [57].

### Morphological data analysis

For *G. fuliginosa*, morphological and weight measurements across different sampling areas were compared using the Kruskal-Wallis Rank Sum test due to the non-normal data distribution. We conducted six tests with morphological measurements (weight, wing length, tarsus length, beak length, beak width, and beak depth) as dependent variables and the four surveyed areas (Urban, Agricultural, Deciduous Forest, and Evergreen Forest) as explanatory variables. If the Kruskal-Wallis test indicated significance (*p* < 0.05), we conducted a post hoc analysis using the Dunn test with Bonferroni p-value correction to assess differences between areas.

For *S. petechia aureola*, analysis of variance tests (ANOVA) were employed for morphological and weight measurements that followed a normal data distribution. The dependent variables were weight, wing length, tarsus length, beak length, beak width, and beak depth, and the four surveyed areas (Urban, Agricultural, Deciduous Forest, and Evergreen Forest) served as explanatory variables. If a test was significant (*p* < 0.05), a Tukey post hoc test was applied to explore differences between areas. If the morphological parameter data did not follow a normal distribution, the analysis described above for *G. fuliginosa* data was employed.

### Corticosterone regression model

We used corticosterone concentration data to build an exploratory linear regression model for each species to examine the relationship between corticosterone concentration in tail feathers and anthropogenic impact. In the model, the continuous outcome variable was corticosterone concentration (ng hormone/mg feather), while the categorical explanatory variables were the surveyed areas and the year of collection. In addition, to account for body weight and size, we included weight and wing length of birds in the model as potential explanatory factors. To ensure the most effective model, we examined the normality and homoscedasticity of the residuals for each model.

For the regression model of *G. fuliginosa*, we divided the deciduous forest area into its three sampling points: Playa Ochoa, Playa Carola, and Playa Loberia. This decision was taken to explore potential differences in birds captured in natural areas near the urban area compared to those further away. Unlike other sampled areas, sampling points within the Deciduous Forest Area are relatively distant from each other and have different proximities to the city (Fig. 1). Deciduous forests at Carola and Loberia are accessible by land from the town of Puerto Baquerizo Moreno and received 24,909 and 19,459 tourists, respectively, in 2018 [58]. In contrast, Playa Ochoa is not listed as a nearby visitor site from Puerto Baquerizo Moreno, requiring a boat ride of approximately 30 min to access. Although visitor reports for Playa Ochoa are unavailable, we assume visitor flow is much lower than at Playa Carola and Loberia due to its restricted access.

Pairwise comparisons of means were performed to explore specific differences in bird corticosterone concentration between areas, using the estimated marginal means with the emmeans package in R. The comparisons were adjusted for multiple testing using the Tukey method with a significance threshold of *p*-value < 0.05.

For the regression model of *S. petechia aureola*, we decided to treat deciduous forests as a single explanatory variable due to low capture rates at some sampling points. Nevertheless, to account for intra sampling point variability, we used a linear mixed-effect model, with corticosterone concentration as the dependent variable and area, weight, and wing length as fixed effect predictors. Sampling points were included as a random effect to account for potential variability within deciduous forests. The model was fitted using the lme4 package in R, with parameter estimation conducted using REML.

## Results

### Morphological analysis

We analysed morphometric data based on 133 individuals of *G. fuliginosa* (103 in 2018 and 30 in 2019, Table 2) and 45 individuals of *S. petechia aureola* (18 in 2018 and 27 in 2019; Table 3).

**Table 2:** Morphometric measurements (average and standard deviation) of Small Ground Finch *G. fuliginosa* captured in four sampling areas of San Cristobal Island, Galapagos, between May and July 2018 and 2019

	Weight (g)	Wing length (cm)	Tarsus length (cm)	Beak length (cm)	Beak width (cm)	Beak depth (cm)
All birds (*n* = 133)	15.48 (2.65)	6.08 (0.42)	2.01 (0.24)	0.88 (0.09)	0.51 (0.09)	0.69 (0.08)
Deciduous Forest (*n* = 73)	15.14 (1.64)	6.01 (0.34)	2.07 (0.21)	0.86 (0.08)	0.51 (0.08)	0.68 (0.04)
Urban Area (*n* = 25)	17.30 (4.17)	5.90 (0.58)	1.93 (0.37)	0.88 (0.09)	0.53 (0.12)	0.70 (0.13)
Seasonal Evergreen Forest (*n* = 11)	15.41 (2.09)	6.38 (0.24)	1.95 (0.07)	0.92 (0.12)	0.53 (0.1)	0.72 (0.11)
Agricultural Area (*n* = 24)	14.63 (2.65)	6.33 (0.35)	1.94 (0.17)	0.89 (0.08)	0.51 (0.09)	0.68 (0.08)

**Table 3:** Morphometric measurements (average and standard deviations) taken of Galapagos Yellow Warbler *Setophaga petechia aureola* captured in four sampling areas of San Cristobal Island, Galapagos, between May and July 2018 and 2019

	Weight (g)	Wing length (cm)	Tarsus length (cm)	Beak length (cm)	Beak width (cm)	Beak depth (cm)
All birds (*n* = 45)	12.45 (1.51)	6.24 (0.35)	2.07 (0.27)	0.95 (0.42)	0.36 (0.03)	0.32 (0.02)
Deciduous forest (*n* = 15)	12.30 (1.94)	6.22 (0.42)	2.16 (0.15)	1.07 (0.72)	0.36 (0.03)	0.32 (0.03)
Urban area (*n* = 11)	13.20 (1.52)	6.21 (0.30)	2.00 (0.27)	0.88 (0.06)	0.34 (0.03)	0.30 (0.02)
Seasonal evergreen forest (*n* = 9)	11.97 (1.09)	6.25 (0.33)	1.95 (0.37)	0.88 (0.03)	0.38 (0.04)	0.32 (0.02)
Agricultural area (*n* = 10)	12.26 (1.51)	6.27 (0.35)	2.13 (0.27)	0.89 (0.42)	0.36 (0.03)	0.31 (0.02)

We found significant differences in the weight of *G. fuliginosa* across the different sampling areas (X^2^ = 10.119, df = 3, *p* < 0.05). Birds captured in the urban area exhibited higher weight compared to those from the agricultural area (*p* < 0.05) and the deciduous forest (*p* < 0.01). We also found significant differences in wing length (X^2^ = 26.12, df = 3, *p* < 0.001). Wing length of birds from the seasonal evergreen forest was significantly greater than those from the urban of area (*p* < 0.001) and the deciduous forest (*p* < 0.001). Additionally, birds from the agricultural area showed higher wing length compared to those from the urban area (*p* < 0.001) and deciduous forest (*p* < 0.001). Tarsus length exhibited significant differences (X^2^ = 10.93, df = 3, *p* > 0.05), with birds from the deciduous forest showing significantly longer tarsus compared to birds from the agricultural area (*p* < 0.05). We did not find significant differences in beak length (X^2^ = 5.47, df = 3, *p* > 0.05), beak width (X^2^ = 0.89, df = 3, *p* > 0.05) or beak depth (X^2^ = 3.14, df = 3, *p* > 0.05) between the areas (Table 2).

For *S. petechia aureola* we did not find significant differences in weight (X^2^ = 5.3, df = 3, *p* > 0.05), tarsus length (X^2^ = 4.33, df = 3, *p* > 0.05), beak length (X^2^ = 1.18, df = 3, *p* > 0.05), wing length (F = 0.063, *p* > 0.05) or beak depth (F = 2.75, *p* > 0.05) between the areas. However, we found a significant difference in beak width (F = 3.37, *p* < 0.05); birds from the seasonal evergreen forest had significantly wider beaks (*p* < 0.05) compared to those from the urban area (Table 3).

### Corticosterone analysis of *G. fuliginosa*

We use feathers from 65 finches captured in 2018 and 2019 for corticosterone analyses. In 2018, we used six samples of finches from the urban area, 17 from the deciduous forest (eight in Playa Ochoa, four in Playa Loberia and five in Playa Carola), 10 from the seasonal evergreen forest, and 10 from the agricultural area. In 2019, due to a reduction in finch abundance and captures, samples were taken from eight finches in the urban area and 14 finches in the deciduous forest (seven in Playa Ochoa, five in Playa Loberia, two in Playa Carola); however, no samples were obtained from the agricultural area and evergreen seasonal forest in that year. The residuals of *G. fuliginosa* better met the conditions of normality and homoscedasticity with a logarithmic transformation of the continuous variable.

The exploratory linear regression model for corticosterone concentration values (R2 = 0.85; *p* < 0.001) predicted for the intercept represents the value for birds captured in Playa Ochoa (deciduous forest) with a weight of 10 g and a wing length of 5 cm, resulting in an exponentiated value between 11.85 ng and 18.92 ng corticosterone/mg feather. The coefficient explaining most of the variability was the year 2019, with an estimated prediction between 65% and 73% reduction in corticosterone concentration compared to the prediction for birds captured in 2018. Changes in weight and wing length were not significant predictors of corticosterone concentration variability (Fig. 4). The detailed results of the regression model for *G. fuliginosa* are presented in Table 4.

**Table 4:** Corticosterone concentration regression results for each predictive variable in the corticosterone concentration regression model for *G. fuliginosa*

Coefficients	Estimate (ngC/mgF)	Exp. estimate	Standard error	T value	***p*** value	Confidence interval (95%)	Exp. confidence interval
Intercept	2.70	14.97	0.12	23.16	<0.001	(2.47/2.94)	(11.85/18.93)
Playa Loberia (deciduous forest/touristic)	0.39	1.47	0.09	3.98	<0.001	(0.19/0.58)	(1.21/1.7 9)
Playa Carola (deciduous forest/touristic)	0.39	1.48	0.1	3.74	<0.001	(0.18/0.60)	(1.20/1.82)
Urban Area	0.43	1.54	0.09	4.88	<0.001	(0.26/0.61)	(1.29/1.85)
Agricultural Area	−0.1	0.9	0.09	−1.03	0.3	(−0.29/0.09)	(0.74/1.09)
Evergreen seasonal forest	−0.35	0.70	0.10	−3.47	<0.001	(−0.55/−0.15)	(0.57/0.86)
Weight	−0.01	1.00	0.01	−0.31	0.19	(−0.03/0.007)	(0.96/1.0)
Wing length	−0.12	0.88	0.09	−1.33	0.07	(−0.3/0.06)	(0.74/1.06)
Year 2019	−1.17	0.31	0.07	−16.82	<0.001	(−1.31/−1.03)	(0.27/0.35)

### Pairwise comparison of *G. fuliginosa* corticosterone concentration means between areas

The regression model and the post hoc pairwise comparisons of corticosterone concentrations among *G. fuliginosa* from different areas revealed several significant differences. The low touristic deciduous forest area Playa Ochoa showed significantly lower corticosterone levels compared to the touristic deciduous forest areas, Playa Carola (estimate = −0.39, SE = 0.10, *p* < 0.01) and Playa Loberia (estimate = −0.38, SE = 0.097, *p* < 0.005), and the Urban Area (estimate = −0.43, SE = 0.089, *p* < 0.001). In contrast, the birds in Playa Ochoa had significantly higher corticosterone levels compared to the evergreen forest area (estimate = 0.35, SE = 0.100, *p* < 0.05), while no significant difference was observed between Playa Ochoa and the agricultural area (*p* = 0.90).

The birds in Playa Carola had significantly higher corticosterone concentrations compared to the birds in the agricultural area (estimate = 0.49, SE = 0.11, *p* < 0.01) and the evergreen forest area (estimate = 0.74, SE = 0.12, *p* < 0.001). No significant differences were found between the values of the two deciduous forest touristic areas Playa Carola and Playa Loberia or the urban area (*p* = 1 and *p* = 0.99, respectively). Nor between Playa Loberia and the urban area (*p* = 0.9960).

**Fig. 2:**
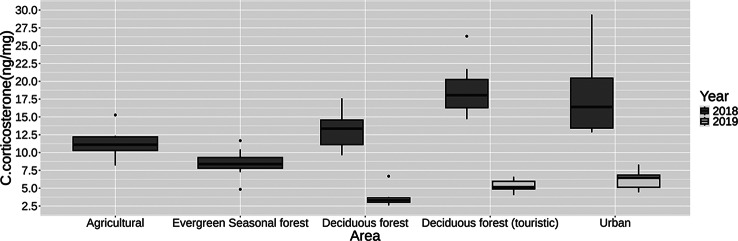
Boxplots representing the corticosterone concentration (ngCort/mgFeather) values for *G. fuliginosa* finches caught in 2018 and 2019 in the four sampling areas. The deciduous forest area results are divided in the ones obtained for the close urban touristic deciduous forest trails Playa Carola and Playa Loberia (deciduous forest touristic) and the ones obtained for the less touristic deciduous forest trail Playa Ochoa (deciduous forest)

The birds in the agricultural area had significantly lower predicted corticosterone levels compared to Playa Loberia (estimate = −0.49, SE = 0.11, *p* < 0.001) and the urban area (estimate = −0.54, SE = 0.11, *p* < 0.001), but not compared to the evergreen forest area (*p* = 0.1608). Lastly, the birds in the evergreen forest area exhibited significantly lower corticosterone levels compared to the urban area (estimate = −0.78443, SE = 0.1105, *p* < 0.0001) and Playa Loberia (estimate = 0.74, SE = 0.11, *p* < 0.001). The differences in corticosterone concentration in the birds of each area can be visualised in Fig. 2.

### Corticosterone analysis of *S. petechia* aureola

For *S. petechia aureola,* we used data from 45 birds captured in 2018 and 2019. In 2018, we collected six samples in the urban area, five in the deciduous forest, three in the seasonal evergreen forest, and four in the agricultural area. In 2019, we analysed samples from five birds from the urban area, 10 birds from the deciduous forest, six from the seasonal evergreen forest, and six from the agricultural area.

The model’s residuals exhibited more or less homoscedasticity and normal distribution that did not improve with data transformation. The exploratory linear mixed-effects model for the corticosterone concentration values predicted for the intercept, birds captured in the deciduous forest in 2018 with a weight of 6 g and a wing length of 1 cm, a value of hormonal concentration between 16.00 and 42.90ng corticosterone/mg feather. The random effects were included to account for the variability within sampling points in the deciduous forest area. The variance component for the random effect was 0.177, and the standard deviation was 0.42.

**Fig. 3:**
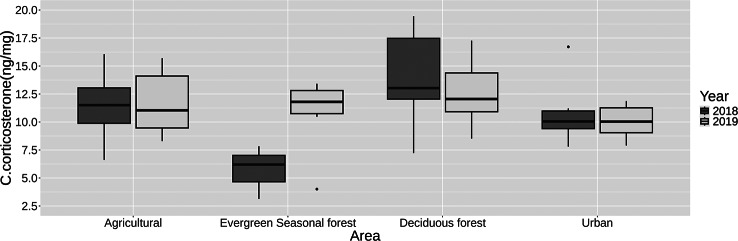
Boxplots representing the corticosterone concentration values (ngCort/mgFeather) for the *S. petechia aureola* caught in 2018 and 2019 in the four sampling areas

Regarding the fixed effects, we found no significant differences associated with the sampling areas. We found a significant reduction in corticosterone concentration associated with wing length; an increase of 1 cm in wing length predicted a reduction in corticosterone concentration between −5.81 and −0.61 units relative to the baseline intercept value. There were no significant variations related to weight or year of collection (Fig. 3). The regression model results for *S. petechia aureola* are provided in Table 5.

**Table 5:** Corticosterone concentration regression results for each predictive variable with the predicted results for the corticosterone concentration regression model for *S. petechia aureola*

Coefficients	Estimate (ngC/mgF)	Standard Error	T value	*P* value	Confidence interval (95%)
Intercept	29.37	7.31	4.015	<0.001	(16.00/42.9)
Urban Area	−2.36	1.45	−1.63	0.48	(−4.86/0.15)
Agricultural Area	−1.15	1.40	−0.82	0.66	(−3.53/1.27)
Evergreen seasonal forest	−3.75	1.44	−2.60	0.4	(−6.23/−1.25)
Weight	−0.03	0.34	−0.08	0.93	(−0.66/0.59)
Wing Length	−3.20	1.42	−2.26	<0.05	(−5.81/−0.61)
Year 2019	0.69	1.02	0.68	0.50	(−1.20/2.53)

## Discussion

We identified hormonal and morphological trends supporting our hypothesis that there are hormonal and morphological variations in birds from areas with contrasting human land uses, particularly evident in the Small Ground Finch, *G. fuliginosa*. A comparison of corticosterone levels from 2018 and 2019 suggests that seasonal climatic changes may have physiological consequences for *G. fuliginosa*. Although we recognize that our sample size does not permit a final conclusion, our results suggest species-specific outcomes regarding the impact of land use changes on the morphology and corticosterone levels of songbirds, as we did not observe signs of these factors influencing the Galapagos Yellow Warbler, *S. petechia aureola*. These findings are relevant since glucocorticoids play a crucial role in vertebrate physiology, affecting immune capacity, nervous development, and reproduction [7].

### Body Measurements

Wild animals in urban environments often show behavioural adaptations in feeding preferences; still, not all populations use resources offered by urbanisation similarly [59], and granivorous species are typically better adapted to urban areas than insectivorous species [60, 61]. In our study, this guild differentiation may explain why the weight of the granivorous *G. fuliginosa* in the urban area was higher than in natural forested and agricultural areas (Table 2). Conversely, no clear differences were found in the weight of the insectivorous *S. petechia aureola.* The diet of *G. fuliginosa* in natural areas consists mainly of small seeds and fruits [62]. However, a study on Santa Cruz Island, Galapagos, suggests that finches in urban areas prefer human-produced food over native seeds [33]. This phenomenon could repeat itself in San Cristobal, with urban finches being more influenced by human food sources and consequently having a different weight from finches in natural areas. The generalist nature of a species’ diet is correlated with its ability to exploit resources in urban habitats more effectively [63]. *Setophaga petechia aureola* has been reported as mainly insectivorous [64], although there are no published comprehensive studies on its diet in the human-populated islands of the Galapagos. We hypothesised that the species is probably less suited for anthropogenic food in urban areas than finches. As a result, our study did not reveal a significant variation in weight between populations living in natural areas and those in urban areas.

Differences attributed to adaptive evolution on microgeographical scales have been documented in inhabited islands of the Galapagos. While our study revealed larger wings in *G. fuliginosa* from the highlands of San Cristobal Island (Table 2), a study in Santa Cruz Island reported that *G. fuliginosa* from the highlands exhibited larger beaks, thicker tarsi, and smaller feet when compared with those from the lowlands [37]. One suggested explanation for morphological disparities between lowland and highland finches in the Galapagos is the adaptation of populations to local resources [37]. Deciduous forest birds have been documented to spend more time foraging for seeds on the ground. In contrast, highland birds in the seasonal evergreen forest tend to forage for fruits and seeds between low vegetation [53]. This differential foraging behaviour could affect flying behaviour and potentially influence wing size. Our findings of smaller tarsi in finches from the deciduous forest compared to those from the agricultural area (Table 2) may also be related to ecological differences between these regions. While our study’s scale does not allow definitive conclusions about evolutionary adaptations, we believe it would be interesting to explore the environmental pressures that could be related to this difference in wing size.

Interestingly, we did not find significant differences in beak size between finches from the deciduous forest and those from the seasonal evergreen forest (Table 2), in contrast to findings reported in populations on Santa Cruz Island [37]. There are several potential explanations for these differences. It is possible that the sampling effort in our study was not sufficient to capture this reported trend. Also, land-use change may affect niche segregation in finches and, therefore, beak size differences between populations. Studies have previously highlighted that feeding opportunities in urban areas of the Galapagos may reduce niche segregation between finch species [33]. Research conducted on *G. fuliginosa* in Santa Cruz Island suggested that individuals in agricultural areas have intermediate beak sizes compared to birds living in highland and lowland natural areas [37]. Given that the natural areas sampled in our study were relatively close to urban and agricultural areas, it is plausible that we did not find beak differences between finches due to a potential decrease in adaptive pressure, possibly represented by changes in land use. Further investigations with expanded sampling efforts and a focus on the dynamic interplay of land use and adaptive pressures could shed light on the intricate relationships influencing beak size variations in Galapagos finch populations.

Interestingly, we found that *S. petechia aureola* from the seasonal evergreen forest exhibited wider beaks than those in the urban area. The interpretation of these results is challenging due to the lack of published studies on the foraging behaviour of these birds in the Galapagos. Browne et al. [65], reported differences in beak morphology in *S. petechia aureola* populations from different archipelago islands. The authors postulated that these variations could be driven by the presence or absence of other insectivorous birds, such as Galapagos Flycatcher *Myiarchus magnirostris* or Grey Warbler-Finch *Certhidea fusca* that share similar diets with *S. petechia aureola* [65]. Both species were present in our study areas on San Cristobal Island. Exploring the interactions and dynamics of these species in the Galapagos Islands could provide valuable insights into the factors shaping beak morphology in insectivorous birds.

### Corticosterone analysis

Our results indicate that *G. fuliginosa* individuals living in the urban area of San Cristobal exhibited higher corticosterone concentration levels than those from natural and agricultural areas. This observation was further supported by the discovery that finches captured in deciduous forests close to the urban area and with higher tourism displayed higher hormone concentration levels than those from the deciduous forest further away from the urban area and with lower tourism (Fig. 2). Various stressors associated with urban areas and tourism, such as noise, light pollution, and dietary changes have been identified to have behavioural and physiological effects on bird populations [66–71]. Studies on Santa Cruz Island have suggested that dietary changes linked to urbanisation and human presence can impact behaviour, gut microbiota, and even epigenetic marks in finches [33, 35, 36]. Our results may indicate that urban land use contributes to increased corticosterone for *G. fuliginosa* populations. Furthermore, the finding that finches from the island lowlands, regardless of land use, had higher hormonal stress levels than those captured in the island highlands suggests that the influence of urban land use extends beyond town limits. The effects of dietary change associated with human presence in the town have also been reported in finches living in natural vegetation areas close to urban areas on Santa Cruz Island [33, 36].

In contrast, we did not find clear differences in hormone stress concentration for *S. petechia aureola* between the different sampling areas (Fig. 3). The effects of urbanisation on hormonal stress in birds are not universal and depend largely on context and species [19, 72, 73]. Our results provide evidence of how urban land use could have different hormonal effects on species of two different guilds. Notably, for *S. petechia aureola*, we identified a relationship between larger wing sizes and lower stress concentrations (Table 4). Although a negative relationship between corticosterone concentration and postnatal growth has been reported for some bird species [74, 75], specific studies on *S. petechia aureola* body condition and hormone concentration during development should be carried out to draw further insights.

Our results indicate that *G. fuliginosa* captured in 2018 had higher stress levels than those captured in 2019 (Fig. 2). A possible explanation for this observation could be the pronounced environmental change between the two years. Birds are known to exhibit elevated stress responses to abrupt environmental changes [76]. Records for February, during the rainy season in San Cristobal Island, show that the precipitation in 2018 (about 240 mm of rainfall) was much higher than the combined rainfall in the same month of the previous three years, where less than 50 mm of rainfall was recorded for February. The 2018 February precipitation was also higher than 2019, which recorded less than 150 mm of rainfall [77–82]. A study of corticosterone levels in Galapagos birds during the 1998 El Niño rainy season and the 1999 La Niña dry season showed much higher corticosterone levels in finches caught in 1998 [83]. Our results seem to align with the trend that unusually rainy seasons imply an increase in hormonal stress in finches in the Galapagos archipelago. We did not find the same for *S. petechia aureola*, where there seems to be no clear influence of year on stress concentrations.

Our findings prompt to consider the importance of the ecology of each species in navigating the dynamics of land use changes and seasonal environmental shifts. A deeper understanding of the ecology and behaviour of Galapagos land bird species, particularly its relation to morphological and hormonal changes, could be fundamental for predicting the impacts of land use, pathogens and climate change on their conservation. We hope that the trends highlighted in this study could serve as a starting framework, paving the way for more extensive research endeavours focused on the health of land birds in the Galapagos archipelago and its intricate connections to stress and human impacts.

## Data Availability

Data is provided within the manuscript.
